# A Rare Presentation of Lymphoma of the Cervix with Cross-Sectional Imaging Correlation

**DOI:** 10.1155/2014/157268

**Published:** 2014-04-17

**Authors:** Brinda Rao Korivi, Corey T. Jensen, Madhavi Patnana, Keyur P. Patel, Tharakeswara K. Bathala

**Affiliations:** Department of Diagnostic Radiology, The University of Texas M. D. Anderson Cancer Center, Pickens Academic Tower, 1400 Pressler Street, Unit 1473, Houston, TX 77030-4009, USA

## Abstract

Non-Hodgkin's lymphoma of the cervix is an extremely uncommon entity, with no standard established treatment protocol. A 43-year-old asymptomatic female with a history of dual hit blastic B-cell lymphoma/leukemia in complete remission presented with an incidental cervical mass, which was initially felt to represent a cervical fibroid on computed tomography (CT). It was further evaluated with ultrasound, biopsy, and positron emission tomography-computed tomography (PET-CT), which demonstrated a growing biopsy-proven lymphomatous mass and new humeral head lesion. The patient was started on chemotherapy to control the newly diagnosed humeral head lesion, which then regressed. She then underwent radiation to the cervix with significant improvement in the cervical lymphoma. A review of cross-sectional imaging findings of lymphoma of the cervix is provided, including how to differentiate it from other more common diseases of the cervix. Clinical awareness of rare cervical masses such as lymphoma is very important in order to achieve timely diagnosis and appropriate treatment.

## 1. Introduction

Lymphoma of the female genital tract is very rare, accounting for less than 0.5% of gynecologic malignancies and 1.5% of extranodal non-Hodgkin's lymphoma [1]. More common sites of non-Hodgkin's lymphoma arise in lymph nodes or lymphatic organs including the spleen and thymus. In 25% to 40% of non-Hodgkin's lymphoma cases, the malignancy arises from an extranodal site and is localized, referred to as primary extranodal lymphoma. The gastrointestinal tract is the most common site of primary extranodal lymphoma, but involvement of almost any organ, including the reproductive tract most commonly the cervix, can also occur [2]. The involvement of the cervix in the setting of multiorgan disease as in this report is still rare but more common than primary lymphoma [3, 4].

Clinical symptoms are nonspecific and may be similar to squamous cervical carcinoma or endometrial adenocarcinoma with pain, vaginal discharge, and bleeding or in this case be asymptomatic. Most cases are incidentally detected by Papanicolaou smear tests, but some are incidentally detected with imaging, as in this case report in which initial detection was with CT [5].

## 2. Case Presentation

**Figure 1:**
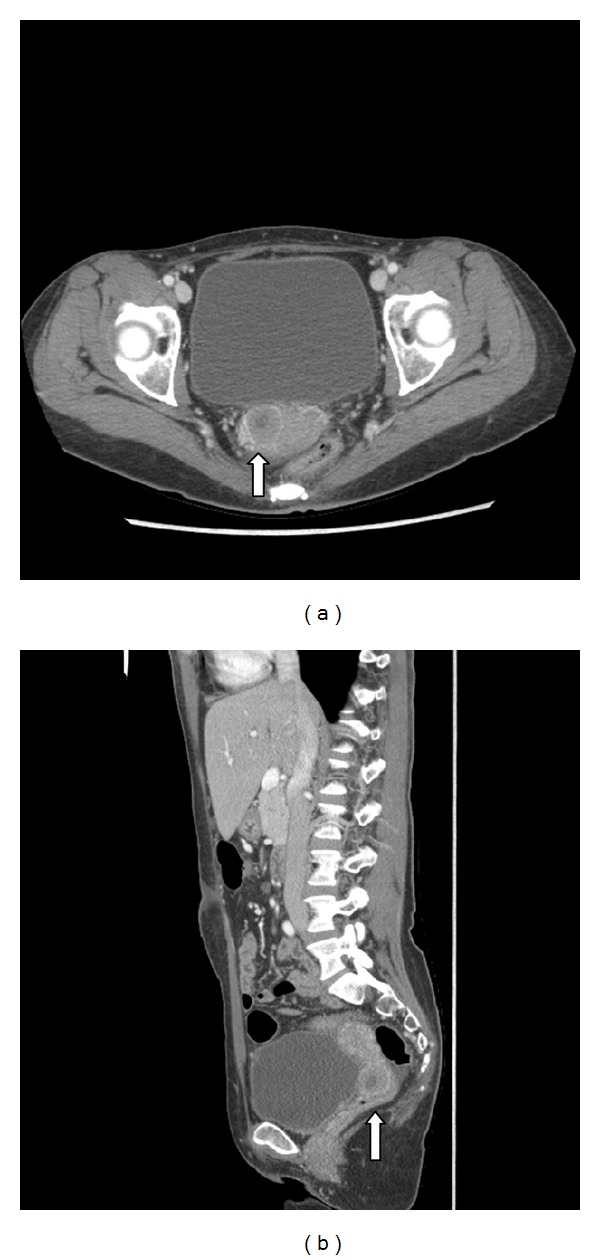
Axial (a) and sagittal (b) postcontrast CT of the pelvis. An enhancing cervical 2.3 cm × 2.2 cm mass (arrow) is on the right side of the cervix.

A 43-year-old female presented to the gynecology clinic with an incidental 2.3 cm × 2.2 cm mass in the cervix detected on CT, which was initially felt to represent a fibroid. She had no gynecologic symptoms. Her recent PET-CT scan from 2 months prior was normal in the cervical region (Figures 1(a) and 1(b)). She had a remote history of de novo dual hit blastic B-cell lymphoma/leukemia with a history of stem cell transplant which was in complete remission.

**Figure 2:**
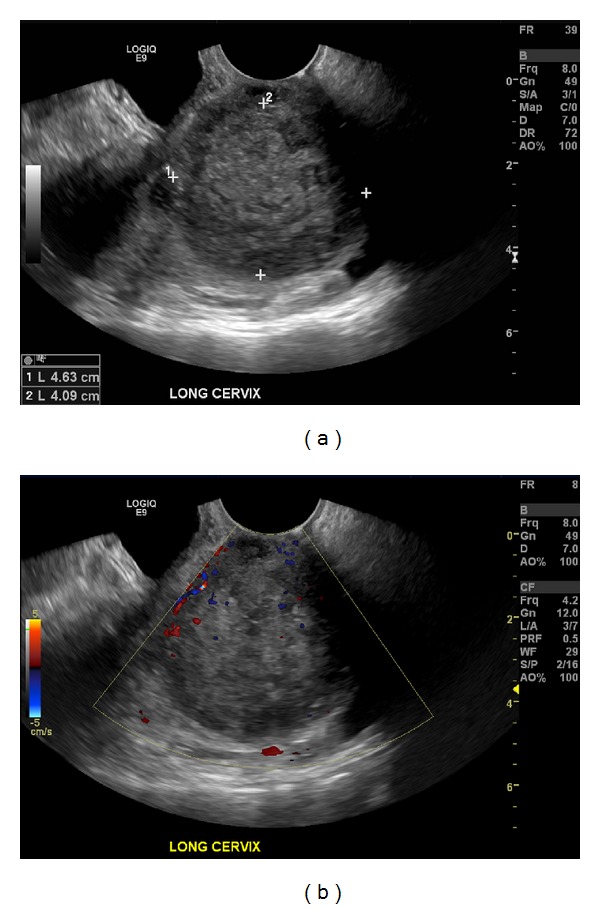
A transvaginal ultrasound. The gray-scale (a) and color Doppler images (b) demonstrate a large heterogeneously echogenic vascular mass measuring 4.6 × 4.1 × 3.6 cm, involving most of the cervix.

A transvaginal ultrasound performed 2 weeks later demonstrated a large heterogeneously echogenic vascular mass measuring 4.6 × 4.1 × 3.6 cm, involving most of the cervix (Figures 2(a) and 2(b)). It increased since the CT performed approximately 1 month before.

**Figure 3:**
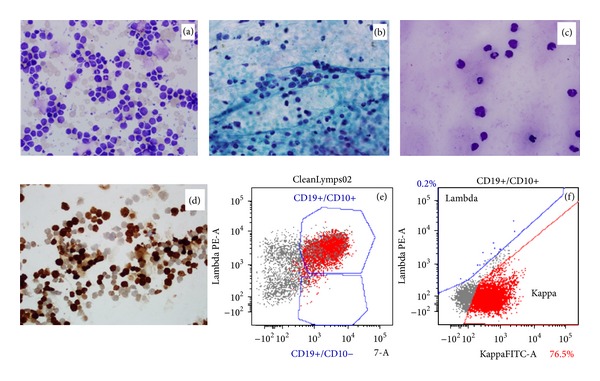
Fine needle aspirate of the cervical mass showed large atypical lymphoid cells in a background of extensive necrosis ((a)–(c)). Representative cytospin (a), aspirate smears Pap stain (b), diff quick stain (c) showing large atypical lymphoid cells with irregular nuclear outlines are shown. Immunoperoxidase stains performed on the cytospins show high Ki-67 proliferation index (d). Immunophenotyping by flow cytometric analysis demonstrates a population of monotypic B-cells expressing CD19, CD10 (e), monotypic kappa light chain (f), and CD38. They are negative for CD5, CD200, CD22, CD43, CD11c, CD23, CD79b, and CD30.

An ultrasound-guided fine needle aspiration (FNA) performed a few days later revealed malignant B-cell lymphoma, consistent with her known history of blastic B-cell lymphoma. The aspirate showed large atypical lymphoid cells in a background of extensive necrosis. Immunophenotyping by flow cytometric analysis demonstrated a population of monotypic B-cells expressing kappa light chain which were positive for CD19, CD10, and CD38 and negative for CD5, CD200, CD22, CD43, CD11c, CD23, CD79b, and CD30. Immunoperoxidase stains (CD3 and Ki-67) performed on cytospin preparations showed a Ki-67 proliferation index of 80%. These findings were consistent with involvement related to the known blastic B-cell lymphoma (Figures 3(a)–3(f)).

**Figure 4:**
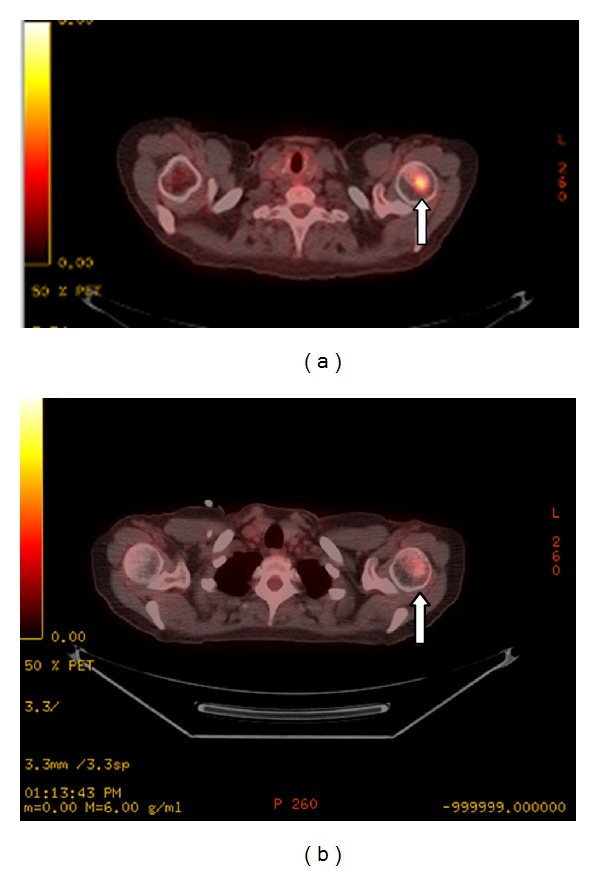
Axial PET-CT fusion image demonstrates an FDG-avid lesion in the left humeral head, consistent with a metastasis, labeled with an arrow (a). Postchemotherapy PET-CT axial image obtained just a few months later demonstrates complete metabolic response to the left humeral head lesion, labelled with an arrow (b).

**Figure 5:**
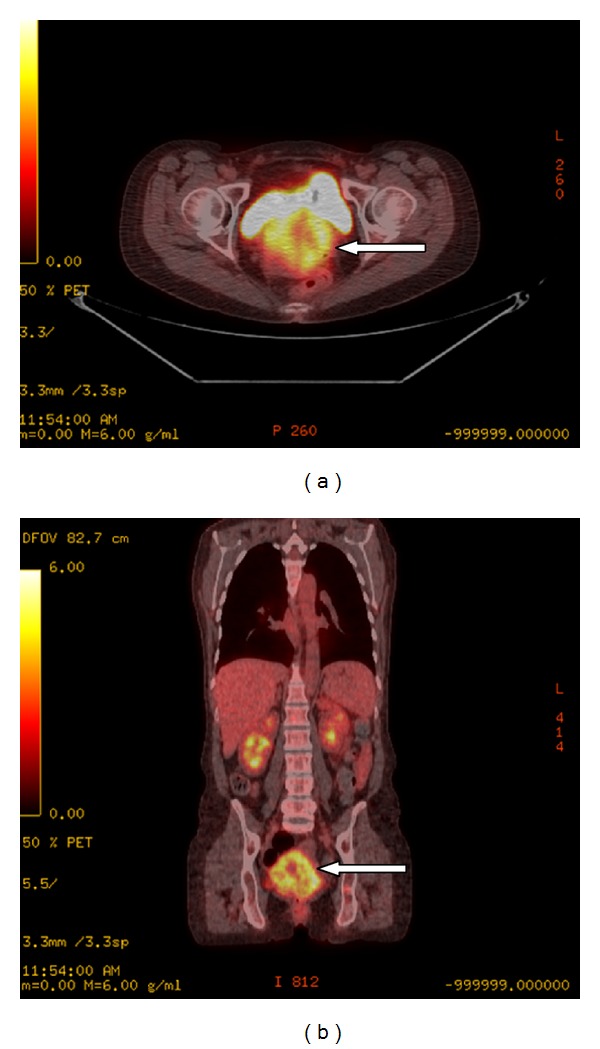
Axial (a) and coronal (b) PET-CT fusion images of the pelvis demonstrate a biopsy-proven lymphomatous 8.4 cm × 5.3 cm mass in the cervix (arrow) with a maximum SUV of 20.7.

A PET-CT obtained 10 days later again demonstrated the new metastatic lesion in the left humeral head (Figure 4(a)). It also depicted the large biopsy-proven lymphomatous cervical mass which had grown since the CT to 8.4 × 5.3 cm, with a maximum standard uptake value (SUV) of 20.7 (Figures 5(a) and 5(b)).

The patient began systemic first salvage chemoimmunotherapy with dose-adjusted EPOCH (etoposide, prednisone, vincristine, cyclophosphamide, and doxorubicin), ofatumumab, dose reductions of bortezomib and continuous infusion of vincristine owing to peripheral neuropathy. The humeral head metastasis significantly improved (Figure 4(b)).

**Figure 6:**
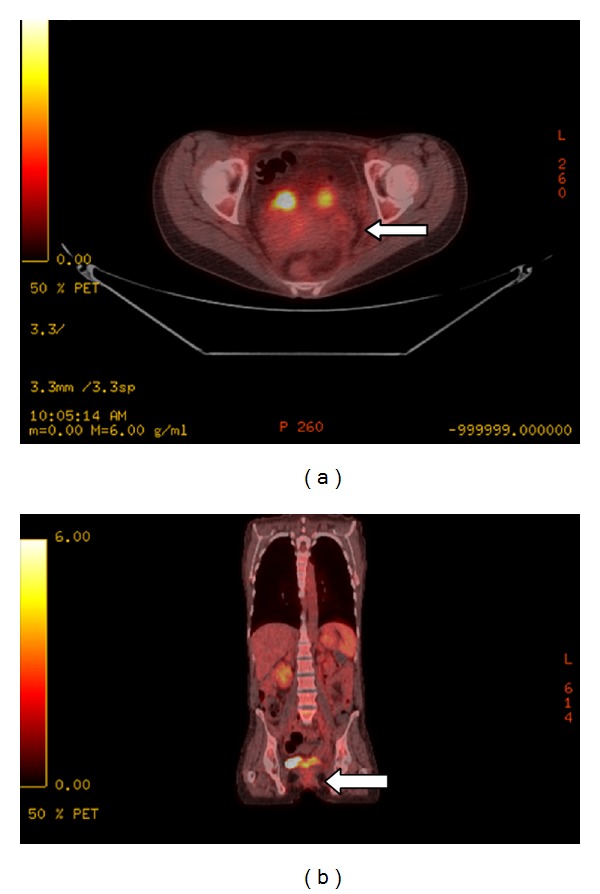
Axial (a) and coronal (b) PET-CT fusion images of the pelvis obtained a few months later after radiation demonstrate significant reduction in the lymphomatous mass (arrow), with no measurable SUV.

The patient then subsequently underwent intensity-modulated radiation therapy (IMRT) to the cervix. The cervical lymphomatous mass subsequently regressed in size, with no measurable abnormal SUV uptake on subsequent PET-CT (Figures 6(a) and 6(b)).

## 3. Discussion

Lymphomas of the female genital tract are very rare. The median age of presentation is 44 years, and the range is from 27 to 80 years. Clinical symptoms are nonspecific and may be similar to squamous cervical carcinoma or endometrial adenocarcinoma, with vaginal bleeding, perineal discomfort, or vaginal discharge [6]. Involvement of the cervix by lymphoma as part of widespread disease can rarely occur, as in this case report.

It is important to be aware of the imaging findings of lymphoma of the cervix as it occurs rarely, and how to differentiate it from more common diseases of the cervix. Cross-sectional imaging modalities including ultrasound, MRI, and PET-CT are useful in the detection of lymphoma in the cervix.

On ultrasound, lymphoma of the cervix presents as a solid mass. The larger lesions tend to be lobulated. The mass typically has abundant vascularity on the Doppler imaging. Other entities to consider in the workup of a cervical mass are fibroids and carcinoma [7]. Fibroids are typically round, exophytic, and relatively less vascular, whereas lymphoma of the cervix is lobulated, expansile, and vascular [8]. Cervical carcinoma typically is slow growing, while lymphoma tends to grow more rapidly and is sometimes not detected on imaging a year before. Also, Papanicolaou smear and human papilloma virus tests can evaluate cervical carcinoma. Ultrasounds are obtained to evaluate the cervix more frequently than MRI due to their widespread availability and cost effectiveness.

MRI is used for tumor delineation with excellent spatial resolution and tissue contrast. An endorectal coil can be useful due to close proximity to the cervix and provides better tissue delineation due to its superior spatial resolution in a relatively small field of view (FOV) [9]. Use of ultrasound gel to distend the cervical canal is also useful for further delineation. In lymphomatous involvement of the cervix, T2 is one of the more useful sequences as lymphoma appears to be a homogeneous, hyperintense mass. On T1-weighted sequences, lymphoma is commonly homogeneous and hypointense. The larger lesions can be infiltrative and lobulated in appearance. They exhibit a strong uniform enhancement pattern on postcontrast T1 imaging, which helps differentiate it from some other entities such as degenerating fibroids, squamous cervical cancer, and endometrial carcinoma. Another differentiating feature is that cervical lymphoma is associated with an intact cervical and endometrial epithelium with considerable involvement of the cervical stroma. These features can differentiate it from cervical carcinoma, in which there is commonly distortion of the mucosa, heterogeneous enhancement, and parametrial invasion [3, 10].

CT imaging is not the modality of choice for evaluating lymphoma of the cervix because the findings are nonspecific with uterine enlargement and lobulated contour, which is also seen commonly with fibroids [9]. It is more useful when CT is combined with PET.

PET-CT is used for localizing and identifying sites of lymphoma which may not be readily apparent on CT. It is also used for identifying lymph node or bone marrow involvement, which is fluorodeoxyglucose- (FDG-) avid. One must be careful to exclude other uterine causes of FDG uptake which include myomas, postpartum state, normal physiologic uptake in the uterus, and other malignancies such as cervical cancer and endometrial cancer [11]. PET-CT can be used to detect recurrent tumor or distant metastases, as in this case in which a distant site of disease was detected in the humerus. It can be used also to assess treatment response as also in this case in which the FDG-avid sites decrease with a favorable treatment response. PET-CT limitations are low spatial resolution of PET and radiotracer excretion artifact in the renal collecting system and bowel [9, 12].

The main differential diagnosis for a cervical mass includes a fibroid and cervical carcinoma. Diagnosis of non-Hodgkin's lymphoma in this case was obtained by direct tissue sampling. Cervical biopsy and immunophenotyping are necessary to differentiate primary cervical non-Hodgkin's lymphoma from benign and malignant disease of the cervix [13–15].

Since B-cell lymphoma of the cervix is such a rare entity, a standard therapeutic regimen has not been established [16]. According to the current literature, treatment options for primary non-Hodgkin's lymphoma of the cervix include chemotherapy, radiation, or radiation combined with either chemotherapy or surgery [17–21]. In cases of relapse, hysterectomy is an option. This patient underwent chemotherapy followed by radiation, which significantly reduced the tumor burden in the cervix.

Awareness of non-Hodgkin's lymphoma of the cervix is important in order to provide timely diagnosis and treatment. Prognosis depends on the lymphoma type and tumor size [22, 23]. Cases tend to have a relapsing and remitting nature; hence, close surveillance including routine pelvic examination, Pap smear, and imaging surveillance in the appropriate clinical setting is required.
